# A Dual Distribution Control Method for Multi-Power Components Energy Output of 4WD Electric Vehicles

**DOI:** 10.3390/s22249597

**Published:** 2022-12-07

**Authors:** Zhiqi Guo, Liang Chu, Zhuoran Hou, Yinhang Wang, Jincheng Hu, Wen Sun

**Affiliations:** 1 State Key Laboratory of Automotive Simulation and Control, Jilin University, No. 5988, Renmin Street, Nanguan District, Changchun 130022, China; 2 Department of Aeronautical and Automotive Engineering, Loughborough University, Leicestershire LE11 3TU, UK; 3 Changzhou Institute of Technology, College of Automotive Engineering, Changzhou 213028, China

**Keywords:** braking energy recovery system, equivalent consumption minimization strategy (ECMS), four-wheel drive (4WD), electric vehicle (EV)

## Abstract

Energy management strategies are vitally important to give full play to the energy-saving of the four-wheel drive electric vehicle (4WD EV). The cooperative output of multi-power components is involved in the process of driving and braking energy recovery of 4WD EV. This paper proposes a novel energy management strategy of dual equivalent consumption minimization strategy (D-ECMS) to improve the economy of the vehicle. According to the different driving and braking states of the vehicle, D-ECMS can realize the proportional control of the energy cooperative output among the multi-power components. Under the premise of satisfying the dynamic performance of the vehicle, the operating points of the power components are distributed more in the high-efficiency range, and the economy and driving range of the vehicle are optimized. In order to achieve the effectiveness of D-ECMS, MATLAB/Simulink is used to realize the simulation of the vehicle. Compared with the rule-based strategy, the economy of D-ECMS increased by 4.35%.

## 1. Introduction

With the shortage of energy and the aggravation of environmental pollution in the world, the use of renewable energy by pure electric vehicles has become the focus of research on zero emission and zero pollution characteristics [1,2,3]. The power motors on the front and rear axles of the four-wheel drive electric vehicle (4WD EV) provide more sufficient power for the vehicle than two-wheel drive electric vehicles (2WD EV) have [4,5]. During braking, the front and rear motors can provide part of the braking torque to realize the braking energy recovery system function of the vehicle [6,7]. However, in the process of vehicle driving and braking, the energy-coordinated output between the front and rear motors directly affects the economy of the vehicle. In order to give full play to the energy-saving potential of 4WD EV multi-power components’ collaborative work, further research on vehicle energy management is required.

In the field of braking research, as a critical technology for energy conservation, a braking energy recovery system can recover part of the energy consumed in the braking process [8,9,10]. In the current research, according to the coupling relationship between motor braking and mechanical braking in the vehicle, the braking modes are divided into parallel types and series types [11,12,13]. The parallel braking energy recovery system directly superimposes the motor braking force on the original friction braking force, which is convenient to implement, but the feedback efficiency is low [14,15]. In contrast, the series braking energy recovery system can prioritize the use of motor braking force and adjust the mechanical braking force accordingly, so that the sum of the two braking forces is consistent with the total braking demand, with higher feedback efficiency and a better braking feeling. Therefore, tandem braking has become the focus and challenge in terms of research [16,17].

In the research on series braking systems, the standard methods are ideal braking force curve distribution [18,19], fixed ratio distribution [20,21,22,23], and optimal front axle braking force distribution [24,25]. The ideal braking force curve distribution strategy distributes according to the ideal braking force curve, which ensures the optimal braking force distribution of the front and rear axles in terms of dynamics, but it is difficult to control the braking pressure of a single axle accurately. The fixed ratio distribution strategy is to select two straight lines as the braking force distribution lines of the front and rear motors with a particular braking strength as the dividing point when it is as close as possible to the ideal braking force distribution curve. This method is simple and practical. Compared with the ideal braking force curve distribution of the front and rear axles, it is easier to achieve. However, this method can only achieve the optimum in a specific operating condition or braking intensity range, and the adaptability to working conditions is poor. The front axle adopts a combination of mechanical braking and motor braking, while the rear axle only has mechanical braking. This method can find a balance between the braking energy recovery system and braking safety, but it does not consider the impact of the braking energy recovery system of the rear motor on the economy. In the 4WD EV, there are some differences in the working characteristics of the front and rear motors. In the series braking mode, because the working state of front and rear motors directly affects the efficiency of energy recovery, on the premise of ensuring the braking stability and the feasibility of the control method, a braking force distribution method for front and rear motors applicable to 4WD EV is developed to improve the efficiency of energy recovery.

In the process whereby the front and rear motors provide the required energy for the vehicle, the proportional distribution of the energy output of the front and rear motors directly affects the economy of the vehicle [26,27]. In the current research, most researchers adopt the rule-based distribution ratio of front and rear motors [28,29], which is simple in structure and easy to implement. However, this strategy has a small scope of application scenarios and poor adaptability to driving conditions. With the further research of experts, optimizing motor working efficiency has become the basis for the energy distribution of front and rear motors [30,31,32]. In order to maximize the efficiency of the front and rear motors, the distribution coefficients of the front and rear motors can be quickly obtained by looking up the table according to different driving conditions. In addition, the vehicle’s power performance and slip rate have also become an essential basis for the energy distribution of front and rear motors, realizing the linear control of the front and rear motors of the whole vehicle [33,34,35]. Under different driving conditions, to further develop the energy-saving potential of 4WD EV, the energy output distribution strategy of front and rear motors has become the focus and challenge of research.

Through the above research and analysis, the power output of front and rear motors directly affects the economy of the vehicle under different driving and braking conditions of 4WD EV. In this context, this paper proposes a method based on the equivalent consumption minimization strategy to achieve multiple solutions for the front and rear motors energy distribution control under different driving conditions of the vehicle, with the primary purpose of economic optimization. According to the actual driving conditions of the vehicle, the optimal energy distribution ratio of the front and rear motors is solved based on meeting the power demand of the vehicle, so that the working points of the front and rear motors are more distributed in the higher efficiency range, giving full play to the energy saving potential of 4WD EV, and improving the economy and driving range of the vehicle.

The innovation points of this paper mainly include the following two points:

1. In the driving and braking process, given the existence of multiple power components in the 4WD EV, the dual equivalent consumption strategy is adopted to realize the energy distribution of the front and rear motors. The economy and driving range of the vehicle are further improved.
2. The braking and driving are integrated under the same control framework to solve multiple controls. At the same time, during the braking process, the maximum braking force of the motor is used to optimize the influence of the motor braking force on the vehicle stability at different vehicle speeds.

## 2. The Studied 4WD EV and Model Construction

### 2.1. The Studied 4WD EV

**Figure 1:**
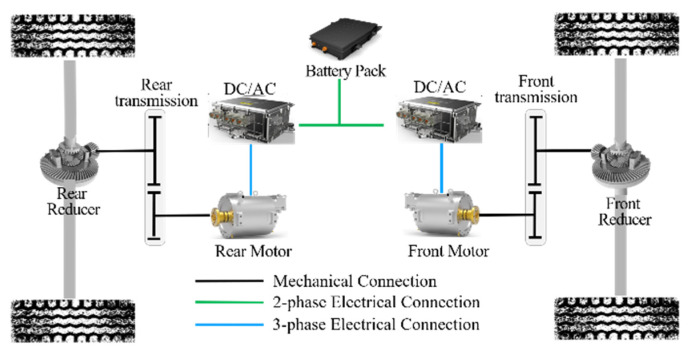
The schematic of the 4DW EV configuration.

This paper mainly takes 4WD EV as the research object. As the power component of the vehicle, the motors are distributed to the front and rear axles. The front and rear motors output power cooperatively to provide power for the vehicle. The structure is shown in Figure 1. Due to the differences in the working characteristics of the front and rear motors, the drive energy distribution of the front and rear motors becomes the key to improving the vehicle’s economy. With the development of braking energy recovery system technology, the reasonable distribution of braking energy of the front and rear motors has become the key technology to improve the economy during the braking process of the vehicle.

Under different driving conditions, according to the working state and economy of power components, this paper can realize three different working modes of the vehicle: front motor drive mode, rear motor drive mode, and dual motor drive mode. The detailed parameters of 4WD EV are shown in Table 1.

### 2.2. Vehicle Dynamic Model

In the 4WD EV research process, the longitudinal stress of the vehicle during driving is considered. In order to ensure normal driving, the vehicle must overcome external resistance, including rolling resistance, air resistance, slope resistance, and acceleration resistance. The relationship between the driving force of the vehicle and the force on the wheel is shown in Equation (1) [36].

(1) $$F_{Wheel}=mgf\cos\theta +\frac{C_{D}Av^{2}}{21.15}+mg\sin\theta +\delta m\frac{dv}{dt}$$

where $F_{Wheel}$ is the tangential driving force generated by the driving wheel; m is the curb weight of the vehicle; g is gravitational acceleration; f is the coefficient of rolling resistance; θ is the road slope; $C_{D}$ is the air resistance coefficient; A is frontal area of the vehicle; δ is the rotational mass conversion factor.

This paper mainly studies the economy of 4WD EV, and does not analyze the vehicle structure in-depth. Therefore, in order to reduce the calculation load, this paper simplifies the complex vehicle model and establishes a simple vehicle longitudinal dynamics model. In order to provide sufficient power for the vehicle to follow the vehicle speed under different driving conditions, the power of components of the vehicle must meet the demand power of the vehicle. In the process of driving and braking, the inertial forces of translation and rotation in the vehicle components should be considered. The equation of the demand power of the vehicle is shown in Equation (2).

(2) $$P_{req}=\frac{mgfv\cos\theta }{3600\eta }+\frac{mgv\sin\theta }{3600\eta }+\frac{C_{D}Av^{2}}{76140\eta }+\frac{\delta mv}{3600\eta }\frac{dv}{dt}$$

where $P_{req}$ is the power demand at wheel side; η is the efficiency of mechanical transmission.

### 2.3. Motor Model

**Figure 2:**
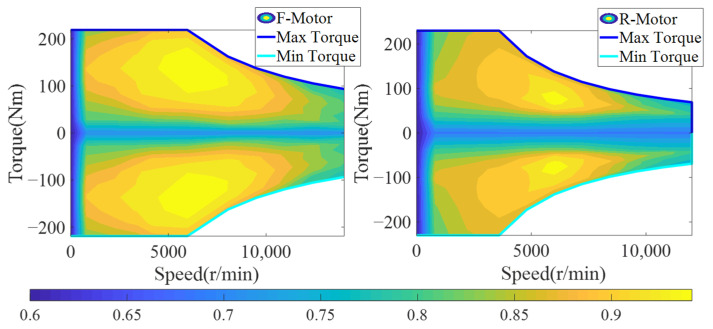
Efficiency maps of front and rear motors.

Permanent magnet synchronous motors (PMSMs) are adopted in the front and rear motors of 4WD EV, which have the advantages of high energy density and efficiency. This paper focuses on the energy distribution of the front and rear motors of the vehicle, and does not do in-depth research on the physical and chemical characteristics of PESMs. Therefore, the dynamic characteristics and thermal performance of PESMs are ignored, and simple motor models are established. The maps of the front and rear motors are established through the calibration of experimental data, as shown in Figure 2.

In the actual driving process of 4WD EV, the front and rear motors drive the vehicle. In addition, the front and rear motors provide counter torque to provide braking force for the vehicle during the braking process of the vehicle. During the driving and braking of the motors, the efficiency of the front and rear motors can be obtained by querying the maps according to information such as speed and torque. According to the different working states of the front and rear motors, the power expressions are shown in Equations (3) and (4).

(3) $$\begin{array}{cc} P_{FM}= & \begin{array}{cc} \{\begin{array}{c} \frac{n_{FM}T_{FM}}{9550\eta_{FM}}\begin{array}{cc} & Driving \end{array}\\ \frac{n_{FM}T_{FM}\eta_{FM}}{9550}\begin{array}{cc} & Braking \end{array} \end{array} & \end{array} \end{array}$$

(4) $$\begin{array}{cc} P_{RM}= & \begin{array}{cc} \{\begin{array}{c} \frac{n_{RM}T_{RM}}{9550\eta_{RM}}\begin{array}{cc} & Driving \end{array}\\ \frac{n_{RM}T_{RM}\eta_{RM}}{9550}\begin{array}{cc} & Braking \end{array} \end{array} & \end{array} \end{array}$$

where $P_{FM}$ is the power of the front motors; $P_{RM}$ is the power of the rear motor; $n_{FM}$ is the speed of the front motor; $n_{RM}$ is the speed of the rear motors; $T_{FM}$ is the torque of the front motor; $T_{RM}$ is the torque of the rear motor; $\eta_{FM}$ and $\eta_{RM}$ are the efficiency of the front and rear motors, respectively.

### 2.4. Battery Model

The battery has very complex physical and chemical characteristics, in which the voltage, current, and internal resistance of the battery are highly nonlinear with the different working conditions of the battery, and the working time of the battery also has a direct impact on the working characteristics. Therefore, it is difficult to establish an accurate model to simulate the working characteristics of the battery.

This paper mainly explores the impact of the energy distribution of the front and rear motors’ driving and braking process on the vehicle economy, not focusing on the internal characteristics of the battery. In order to reduce the calculation load, a simple first-order RC battery model [37] is established in this paper. The current and state of charge (SOC) change rate equations in the battery are shown in Equations (5) and (6).

(5) $$I_{B}=\frac{V_{OC}-\sqrt{V_{OC}{}^{2}-4P_{B}R_{B}}}{2R_{B}}$$

(6) $$S\dot{O}C=-\frac{I_{B}}{Q_{B}}=-\frac{V_{OC}-\sqrt{V_{OC}^{2}-4P_{B}R_{B}}}{2R_{B}Q_{B}}$$

where $I_{B}$ is the current of the battery; $P_{B}$ is the power of the battery; $V_{OC}$ is the open-circuit voltage of the battery; $R_{B}$ is the internal resistance of the battery; $Q_{B}$ is the capacity of the battery.

**Figure 3:**
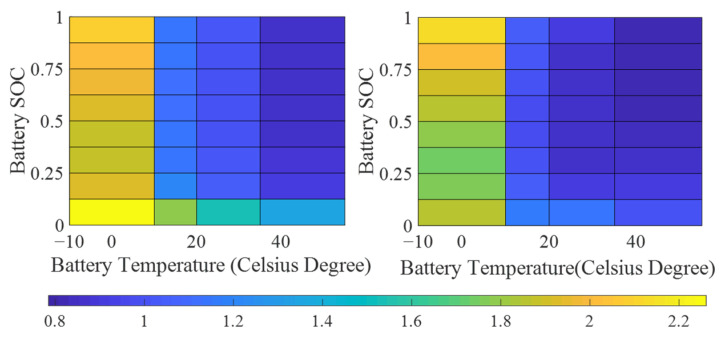
The battery of discharging/charging internal resistance diagrams.

The internal resistance of the battery is directly related to the temperature and SOC of the battery. In this paper, through the calibration of experimental data, data tables of the relationship among the battery internal resistance, battery temperature, and SOC are established, as shown in Figure 3.

## 3. Methodology

**Figure 4:**
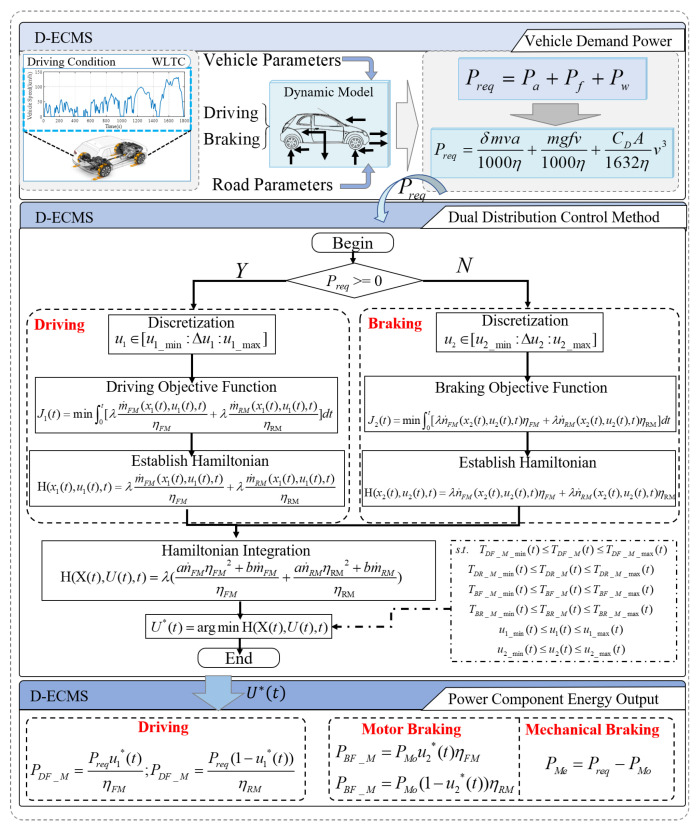
Illustration of the energy management strategy process.

The collaborative output of multi-power components in 4WD EV has a direct impact on the economy of the vehicle. At the same time, in the process of the braking energy recovery system, the braking force distribution of front and rear motors also directly affect the economy of the vehicle. In order to improve the economy of the vehicle and increase the driving range of 4WD EV, this paper adopts the equivalent consumption minimization strategy to integrate the distribution strategy of front and rear motors, under driving and braking conditions, to achieve multiple distributions of energy under different states of the vehicle and improve the economy and driving range of the vehicle. The control architecture is shown in Figure 4. Firstly, in the process of vehicle driving, the required power of the vehicle is calculated according to the pedal position. Secondly, under the condition of meeting the demand power of the vehicle, an equivalent consumption minimization strategy is adopted to realize the coordinated energy output of the front and rear motors. In the braking process, an equivalent consumption minimization strategy is adopted to realize the braking energy distribution of the front and rear motors, realize the optimal management of the front and rear motors, and thus improve the economy of the vehicle. The equivalent consumption minimization strategy is adopted to convert the consumed electricity into the consumed cost (RMB) through the equivalence factor, realizing the dual control of vehicle driving and braking distribution strategies and improving the economy and driving range of 4WD EV.

### 3.1. Driving Energy Distribution Strategy of Front and Rear Motors Based on the Equivalent Consumption Minimization Strategy

The equivalent consumption minimization strategy converts the parameters into evaluable indicators through equivalent factors as an instantaneous optimization strategy. This paper mainly studies the economy of the 4WD EV vehicle, and the electric energy provided by the battery is the only power source of the vehicle. The electricity consumed by the vehicle is the standard for evaluating the vehicle economy. In the process of control strategy construction, in order to better quantify the electricity consumption value, the electricity consumed by the vehicle is converted into electricity cost (RMB) through the equivalence factor, and the energy distribution strategy of front and rear motors is carried out to improve the economy and driving range of 4WD EV.

In 4WD EV, the front and rear motors are coupled with the ground, and the speed of the front and rear motors is proportional to the vehicle speed, as shown in Equations (7) and (8).

(7) $$n_{FM}=\frac{60v}{3.6*(2*\Pi R_{Wheel})}i_{F}$$

(8) $$n_{RM}=\frac{60v}{3.6*(2\Pi R_{Wheel})}i_{R}$$

where v is the speed of the vehicle, km/h; Π is the pi; $R_{Wheel}$ is the wheel radius; $i_{F}$ is the transmission ratio of the front motor; $i_{R}$ is the transmission ratio of the rear motor.

Since there are some differences between the map of the front and rear motors, the efficiency of the front and rear motors working points also significantly affect the efficiency and then affect the economy of the vehicle. An equivalent consumption minimization strategy control strategy is adopted to realize the energy distribution of the front and rear motors, so the working points of the front and rear motors are distributed in the higher efficient range to optimize the economy of the vehicle. The cost of equivalent electricity is shown in Equation (9).

(9) $${\dot{m}}_{equ}(x_{1}(t),u_{1}(t),t)=\lambda \frac{{\dot{m}}_{FM}(x_{1}(t),u_{1}(t),t)}{\eta_{FM}}+\lambda \frac{{\dot{m}}_{RM}(x_{1}(t),u_{1}(t),t)}{\eta_{\mathrm{RM}}}$$

where ${\dot{m}}_{equ}(x_{1}(t),u_{1}(t),t)$ is the electricity charge; λ is equivalence factor; ${\dot{m}}_{FM}(x_{1}(t),u_{1}(t),t)$ is the electricity consumption of front motor; ${\dot{m}}_{RM}(x_{1}(t),u_{1}(t),t)$ is the power consumption of rear motor.

In Equation (9), $x_{1}(t)$ represents the required power of the vehicle at time t as a state quantity and the distribution proportion of the power output of the front and rear motors as a controlled quantity. The output power calculation equation of the front and rear motors is shown in Equation (10).

(10) $$\begin{array}{l} \left\{ \begin{array}{l} P_{FM}(t)=P_{req}(t)*u_{1}(t)\\ P_{RM}(t)=P_{req}(t)*(1-u_{1}(t)) \end{array}\right.\\ \begin{array}{cc} subject & to \end{array}:\\ P_{FM\_\min}(t)\leq P_{FM}(t)\leq P_{FM\_\max}(t)\\ P_{RM\_\min}(t)\leq P_{RM}(t)\leq P_{RM\_\max}(t)\\ u_{1\_}{}_{\min}(t)\leq u_{1}(t)\leq u_{1}{}_{\_\max}(t) \end{array}$$

where $P_{FM}(t)$ is the driving power of the front motor at t time; $P_{RM}(t)$ is the driving power of the rear motor at t time; $P_{req}(t)$ is the required driving power of the vehicle at t time; $P_{FM\_\min}(t)$ and $P_{FM\_\max}(t)$ are the maximum and minimum values of front motor power at t time, respectively; $P_{RM\_\min}(t)$ and $P_{RM\_\max}(t)$ are the maximum and minimum values of rear motor power at t time, respectively; $u_{1\_\min}(t)$ and $u_{1\_\max}(t)$ are the minimum and maximum values of the control variables at time t, respectively.

In the process of establishing the ECMS model, $P_{req}$ is solved through the vehicle dynamics model as the vehicle demand power. This paper mainly studies the impact of 4WD EV motors energy distribution on the vehicle economy. In order to reduce the calculation load of the vehicle dynamics model, the impact of slope on the vehicle is not considered. The vehicle is only affected by rolling resistance, acceleration resistance, and air resistance on flat and straight roads. The equation for solving the required power of the vehicle is shown in Equation (11).

(11) $$P_{req}=P_{a}+P_{f}+P_{w}=\frac{\delta mva}{1000\eta }+\frac{mgfv}{1000\eta }+\frac{C_{D}A}{1632\eta }v^{3}$$

where $P_{req}$ is the demand power of the vehicle; $P_{a}$ is the power of the acceleration resistance; $P_{f}$ is the power of the rolling resistance; $P_{w}$ is the power of the air resistance; v is the vehicle speed; a is the acceleration; η is the total efficiency of the front and rear motors.

The objective function is established to solve the optimal control quantity $u_{1}$ and vehicle economy, as shown in Equation (12).

(12) $$J_{1}(t)=\min\int_{0}^{t}[{\dot{m}}_{equ}(x_{1}(t),u_{1}(t),t)]dt=\min\int_{0}^{t}[\lambda \frac{{\dot{m}}_{FM}(x_{1}(t),u_{1}(t),t)}{\eta_{FM}}+\lambda \frac{{\dot{m}}_{RM}(x_{1}(t),u_{1}(t),t)}{\eta_{\mathrm{RM}}}]dt$$

In vehicle driving, the vehicle status and the information of components are discrete data. Therefore, the Hamilton function is established by Equation (12) to better solve the control variable $u_{1}$ and make the objective function reach the minimum value. The Hamilton function is shown in Equation (13).

(13) $$H(x_{1}(t),u_{1}(t),t)=\lambda \frac{{\dot{m}}_{FM}(x_{1}(t),u_{1}(t),t)}{\eta_{FM}}+\lambda \frac{{\dot{m}}_{RM}(x_{1}(t),u_{1}(t),t)}{\eta_{\mathrm{RM}}}$$

Through Equation (13), the optimal control quantity $u_{1}{}^{*}(t)$ is solved in a finite set, and its expression is shown in Equation (14).

(14) $$\begin{array}{l} u_{1}{}^{*}(t)=\arg\min H(x_{1}(t),u_{1}(t),t)\\ \begin{array}{cc} subject & to: \end{array}\\ T_{DF\_M\_\min}(t)\leq T_{DF\_M}(t)\leq T_{DF\_M\_\max}(t)\\ T_{DR\_M\_\min}(t)\leq T_{DR\_M}(t)\leq T_{DR\_M\_\max}(t)\\ u_{1\_}{}_{\min}(t)\leq u_{1}(t)\leq u_{1}{}_{\_\max}(t) \end{array}$$

where $T_{DF\_M}(t)$ is the driving torque of the front motor at t time; $T_{DR\_M}(t)$ is the driving torque of the rear motor at t time; $T_{DF\_M\_\min}(t)$ and $T_{DF\_M\_\max}(t)$ are the maximum and minimum values of front motor driving torque at t time, respectively; $T_{DR\_M\_\min}(t)$ and $T_{DR\_M\_\max}(t)$ are the maximum and minimum values of rear motor driving torque at t time, respectively.

### 3.2. Vehicle Braking Strategy Based on Series

In this paper, the 4WD EV braking system adopts a series mode, in which the motor braking energy recovery system and the mechanical braking of the vehicle constitute a series braking system. As shown in Equation (15).

(15) $$B_{req}=B_{rec}+B_{mec}$$

where $B_{req}$ is the braking force required by the vehicle; $B_{rec}$ is the brake energy recovery; $B_{mec}$ is the mechanical braking force.

According to Equation (15), the torque provided by the motor and the mechanical braking cooperatively output the braking force to complete the braking demand during the braking process of the vehicle. In the braking process of the vehicle, the change rate of the brake pedal is large, so the vehicle needs to be emergency braked. When the vehicle is in a state of rapid deceleration, mechanical braking is the first to work because of its fast response. With the increase of motor braking force, the mechanical braking decreases accordingly. However, when the vehicle is not in a state of rapid deceleration and the change rate of brake pedal is small, the motor braking plays a significant role. If the motor braking does not meet the braking requirements of the vehicle, the mechanical braking starts to provide the remaining braking force.

**Figure 5:**
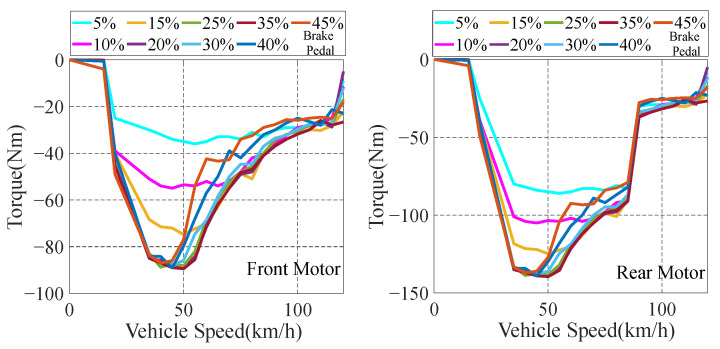
The curves of maximum braking force of the motors under different speed and deceleration pedal.

Considering the stability of the vehicle and the efficiency of the motors, the maximum braking torque provided by the motors varies with the vehicle speed and the opening of the deceleration pedal. Through the calibration of experimental data, the relationship diagrams of the maximum braking force of the motors under different vehicle speed and the deceleration pedal are established, as shown in Figure 5.

According to Figure 5, when the vehicle is at a low-speed state, the braking energy recovery system efficiency of the motors is low, so the motors provide less braking force. When the vehicle is at a high-speed state, the large braking force provided by the motor braking affects the stability of the vehicle. In order to ensure the stability of the vehicle at a high-speed state, the motors still provide less braking force.

### 3.3. The Braking Energy Recovery System Strategy of Front and Rear Motors Based on the Equivalent Minimum Consumption Strategy

Due to the difference between the front and rear motor maps of 4WD EV, the working efficiency of the motors directly affects the efficiency of the braking energy recovery system during the vehicle braking process. This paper adopts an equivalent minimum consumption strategy to distribute the braking energy of the front and rear motors. In the case of meeting the braking demand, the efficiency of the braking energy recovery system is improved, and the driving range of the vehicle is increased.

The equivalent minimum consumption strategy is adopted to establish the front and rear motors’ braking force distribution model to improve the working efficiency of the front and rear motors, optimize the overall braking efficiency of the vehicle, and improve the economy and driving range of the vehicle. The objective function equation is established as shown in Equation (16).

(16) $$J_{2}(t)=\min\int_{0}^{t}[{\dot{n}}_{equ}(x_{2}(t),u_{2}(t),t)]dt=\min\int_{0}^{t}[\lambda {\dot{n}}_{FM}(x_{2}(t),u_{2}(t),t)\eta_{FM}+\lambda {\dot{n}}_{RM}(x_{2}(t),u_{2}(t),t)\eta_{\mathrm{RM}}]dt$$

where ${\dot{n}}_{equ}(x_{2}(t),u_{2}(t),t)$ is the recovery of electric energy; ${\dot{n}}_{FM}(x_{2}(t),u_{2}(t),t)$ is the electric energy recovery of front motor; ${\dot{n}}_{RM}(x_{2}(t),u_{2}(t),t)$ is the electric energy recovery of rear motor.

In Equation (16), $x_{2}(t)$ as a state quantity represents the required braking energy of the vehicle at time t, and $u_{2}(t)$ as a controlled quantity represents the distribution proportion of braking energy of front and rear motors at time t. The braking energy calculation of the front and rear motors is shown in Equation (17).

(17) $$\begin{array}{l} \left\{ \begin{array}{l} B_{FM}(t)=B_{req}(t)u_{2}(t)\\ B_{RM}(t)=B_{req}(t)(1-u_{2}(t)) \end{array}\right.\\ \begin{array}{cc} subject & to: \end{array}\\ B_{FM\_\min}(t)\leq B_{F\_M}(t)\leq B_{FM\_\max}(t)\\ B_{RM\_\min}(t)\leq B_{R\_M}(t)\leq B_{RM\_\max}(t)\\ u_{2\_\min}(t)\leq u_{2}(t)\leq u_{2\_\max}(t) \end{array}$$

where $B_{FM}(t)$ is the braking power of the front motor at t time; $B_{RM}(t)$ is the braking power of the rear motor at t time; $B_{FM\_\min}(t)$ and $B_{FM\_\max}(t)$ are the maximum and minimum values of front motor braking power at t time, respectively; $B_{RM\_\min}(t)$ and $B_{RM\_\max}(t)$ are the minimum and maximum values of rear motor braking power at t time, respectively; $u_{2\_\min}(t)$ and $u_{2\_\max}(t)$ are the minimum and maximum values of the control variables at time t, respectively.

In order to reduce the calculation load, the energy distribution controls of vehicle driving and braking are integrated, and the objective function of the front and rear motors’ distribution strategy is established by Equations (12) and (16), as shown in Equation (18).

(18) $$J(t)=J_{1}(t)+J_{2}(t)\min\int_{0}^{t}[\lambda (\frac{a{\dot{n}}_{F\_M}\eta_{F\_M}{}^{2}+b{\dot{m}}_{F\_M}}{\eta_{F\_M}}+\frac{a{\dot{n}}_{R\_M}\eta_{R\_M}{}^{2}+b{\dot{m}}_{R\_M}}{\eta_{R\_M}})]dt$$

where a and b are constants. when the vehicle is in the braking state, a=0,b=1; when the vehicle is in the driving state, a=1,b=0.

In order to solve the control quantity $U(t)=[u_{1}(t),u_{2}(t)]$ and make Equation (18) reach the minimum value, the Hamilton function is established, as shown in Equation (19).

(19) $$H(X(t),U(t),t)=\lambda (\frac{a{\dot{n}}_{F\_M}\eta_{F\_M}{}^{2}+b{\dot{m}}_{F\_M}}{\eta_{F\_M}}+\frac{a{\dot{n}}_{R\_M}\eta_{R\_M}{}^{2}+b{\dot{m}}_{R\_M}}{\eta_{R\_M}})$$

The optimal control quantity $U^{*}(t)$ is solved in a finite set to make Equation (18) reach the minimum value, and the function is shown in Equation (20).

(20) $$\begin{array}{l} U^{*}(t)=\arg\min H(X(t),U(t),t)\\ \begin{array}{cc} subject & to: \end{array}\\ T_{DF\_M\_\min}(t)\leq T_{DF\_M}(t)\leq T_{DF\_M\_\max}(t)\\ T_{DR\_M\_\min}(t)\leq T_{DR\_M}(t)\leq T_{DR\_M\_\max}(t)\\ T_{BF\_M\_\min}(t)\leq T_{BF\_M}(t)\leq T_{BF\_M\_\max}(t)\\ T_{BR\_M\_\min}(t)\leq T_{BR\_M}(t)\leq T_{BR\_M\_\max}(t)\\ u_{1\_}{}_{\min}(t)\leq u_{1}(t)\leq u_{1}{}_{\_\max}(t)\\ u_{2\_}{}_{\min}(t)\leq u_{2}(t)\leq u_{2}{}_{\_\max}(t) \end{array}$$

where $T_{BF\_M}(t)$ is the braking torque of the front motor at t time; $T_{BR\_M}(t)$ is the braking torque of the rear motor at t time; $T_{BF\_M\_\min}(t)$ and $T_{BF\_M\_\max}(t)$ are the maximum and minimum values of front motor braking torque at t time, respectively; $T_{BR\_M\_\min}(t)$ and $T_{BR\_M\_\max}(t)$ are the maximum and minimum values of rear motor braking torque at t time, respectively.

In order to make the front and rear motors have more operating points distributed in the high-efficiency range during the vehicle driving and braking process and take into account the series braking characteristics of the whole vehicle and the stability of the whole vehicle, an equivalent minimum consumption strategy is adopted to realize the energy distribution of the front and rear motors. This novel method integrates the distribution strategies of driving and braking, realizing the multiple distributions of the energy of the front and rear motors in different states and improving the economy and driving range of the vehicle.

## 4. Simulation Results and Analysis

In vehicle economy analysis, SOC can directly display the degree of vehicle power consumption. Therefore, this paper adopts SOC as the direct evaluation standard of vehicle economy. In order to verify the effectiveness of the dual energy distribution strategy of driving and braking proposed in this paper, a 4WD EV vehicle model is established through MATLAB/Simulink (R2018a), and simulation verification is carried out under the World Light Vehicle Test Cycle (WLTC) driving condition. The practical information on vehicle components in the simulation is collected under four different control strategies, and the data is processed and analyzed. The four different control strategies are described in Table 2. In order to further analyze the impact of different control strategies on the economy of vehicle driving and braking, the working conditions of vehicle components are compared and analyzed. The simulation results show that the novel strategy of driving and braking can effectively improve the vehicle economy and driving range. Please note that the simulation is performed on a computer equipped with an Inteli5-6300HQ processor and 8 GB memory.

### 4.1. The Change and Analysis of SOC under Different Control Strategies

**Figure 6:**
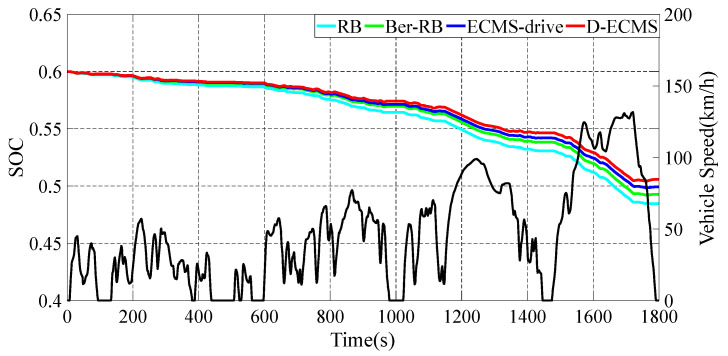
Variation curves of SOC under different strategies.

In the actual driving process of the vehicle, the front and rear motors show different working states with different vehicle demand power and speed. The working efficiency of the motors directly affects the economy of the whole vehicle. The battery is the only power source of the vehicle. As an essential parameter of the battery, SOC directly reflects the economy of the vehicle. In order to analyze the impact of different controls on vehicle economy, the change curve of SOC is shown in Figure 6.

As shown in Figure 6, the curves of SOC show different trends under different control strategies. Based on RB and Bra-RB strategies, the fixed proportion of energy output is adopted. The working state of motors cannot be adjusted according to the driving condition information and the state of the vehicle. The adaptability to driving conditions is relatively poor, resulting in a relatively large decline in the SOC change curve and high-power consumption. Based on ECMS-drive and D-ECMS, the energy distribution of front and rear motors is adopted in the whole vehicle driving process, and the decrease of SOC is relatively small. Compared with RB and Bra-RB strategies, ECMS-drive and D-ECMS show better economies.

The RB strategy does not adopt a braking energy recovery system, and the SOC decreases the most. Thus, the economy is the worst among the four control strategies. Based on the Bra-RB strategy, a fixed proportion of braking energy recovery system of front and rear motors is adopted in the whole vehicle braking process, which is more economical than the RB strategy. Based on ECMS-driver, the energy distribution of front and rear motors is adopted in the driving process. However, the fixed proportion front and rear motor distribution method is adopted in the braking process, which is more economical than the RB and Bra-RB strategies. Based on D-ECMS, the distribution strategy of the front and rear motors is adopted in the driving and braking process, and the decrease of SOC is the smallest. Compared with ECMS-drive, D-ECMS shows the better economy of the vehicle.

As shown in Table 3, compared with the RB strategy, the economic efficiency of D-ECMS is increased by 4.35%, which has better economic optimization. However, the economy improved by 3.01% based on ECMS-drive. Compared with the strategy based on RB, the economy of the Bra-RB strategy is increased by 1.69%. Therefore, the braking energy recovery system can effectively improve the economy of the vehicle and the driving range. Compared with Ber-ECMS, ECMS-driver strategy improves the economy by 1.30%. In the driving process of vehicle, the energy distribution of the front and rear motors can optimize the economy. Compared with D-ECMS and the ECMS-driver, the effect of improving the vehicle economy during the braking process is significant. Therefore, in the braking process, the energy distribution of front and rear motors can further improve the vehicle economy and driving range.

Based on D-ECMS, the dual energy distribution of front and rear motors under the driving and braking state of the vehicle is realized, which can effectively improve the economy of the vehicle. In order to further explore the influence of the working characteristics of the vehicle components on the vehicle economy under different control strategies, it is necessary to analyze the working state of the front and rear motors.

### 4.2. Qualitative Comparison and Analysis of Front and Rear Motors

**Figure 7:**
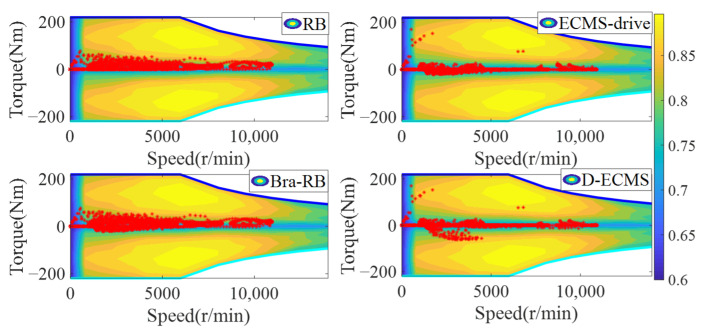
Front motor operating points of energy management strategies.

**Figure 8:**
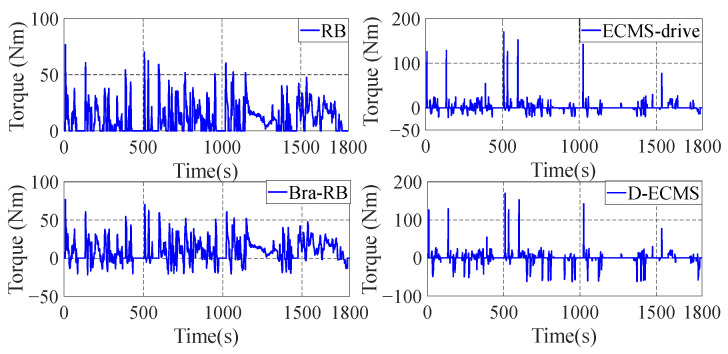
Front motor torque of energy management strategies.

In order to further analyze the influence of the working state of the front and rear motors on the economy in the process of driving and braking, the working point distribution and output torque of the front and rear motors are compared and analyzed. The working state diagram of the front motor is shown in Figure 7 and Figure 8.

As shown in Figure 7 and Figure 8, the working state of the front motor presents different states under different control strategies. The RB strategy has no braking energy recovery system, so the front motor only provides positive torque. Based on the Bra-RB strategy, a negative torque appears which has the function of braking energy recovery, and the operating points are mostly distributed in the low-efficiency interval. Based on the ECMS-driver, the energy distribution strategy of front and rear motors are adopted in the vehicle braking process. As can be seen from Figure 7, more positive torque operating points are distributed in a higher efficiency range. Based on D-ECMS, more front motor braking operating points are distributed in the higher range from 2000 r/min to 5000 r/min. The operating points in the efficiency range can improve the economy of the vehicle and increase the driving range.

**Figure 9:**
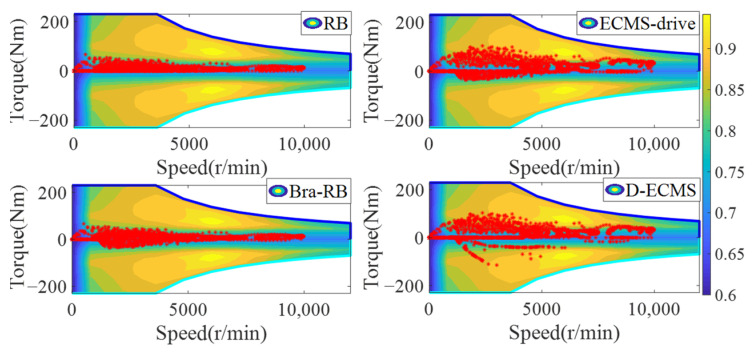
Rear motor operating points of energy management strategies.

**Figure 10:**
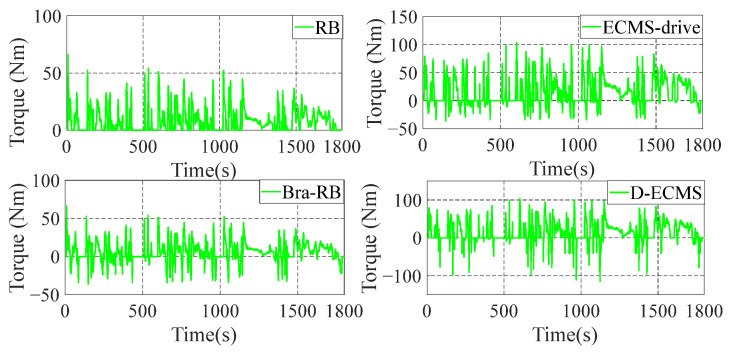
Rear motor torque of energy management strategies.

In order to further analyze the operating points distribution and torque output of the rear motor under different control strategies, the working state diagram of the rear motor is shown in Figure 9 and Figure 10.

As shown in Figure 9 and Figure 10, there is no braking energy recovery system in the vehicle based on RB strategy, and the rear motor only provides positive torque. Under the Bra-RB strategy, the rear motor recovers braking energy by generating negative torque, and the operating points are also distributed in the lower efficiency range. Based on the ECMS-driver, the energy distribution control of the front and rear motors is carried out during the vehicle driving process. After 2000 r/min, the operating points of the rear motors are more distributed in the higher efficiency range, and the improvement effect is significant. Compared with ECMS-drive, D-ECMS adopts the energy distribution strategy of front and rear motors in the driving process, and more braking operating points of the rear motor are distributed in a higher efficiency range, which is more significant from 2000 r/min to 7000 r/min. Under the D-ECMS, the working efficiency of the rear motor is greatly optimized, and the economy and driving range of the vehicle are further improved.

According to Figure 9 and Figure 10, the output torque of front and rear motors varies significantly under different control strategies. Under the ECMS-driver and D-ECMS, the output torque of the front motor in the driving process is small, while the torque output of the rear motor is increased. In the driving and braking process, according to different driving conditions and the energy distribution output of the front and rear motors, the vehicle prefers the output energy of the rear motor. The fundamental reason is that there are some differences in the map of the front and rear motors of the vehicle, and the maximum torque provided by the front and rear motors at different vehicle speeds also presents different characteristics. In order to further analyze the efficient distribution of the front and rear motors under different strategies, analysis and comparison are indispensable.

### 4.3. Quantitative Comparison and Analysis of the Efficiency Distribution of the Front and Rear Motors

**Figure 11:**
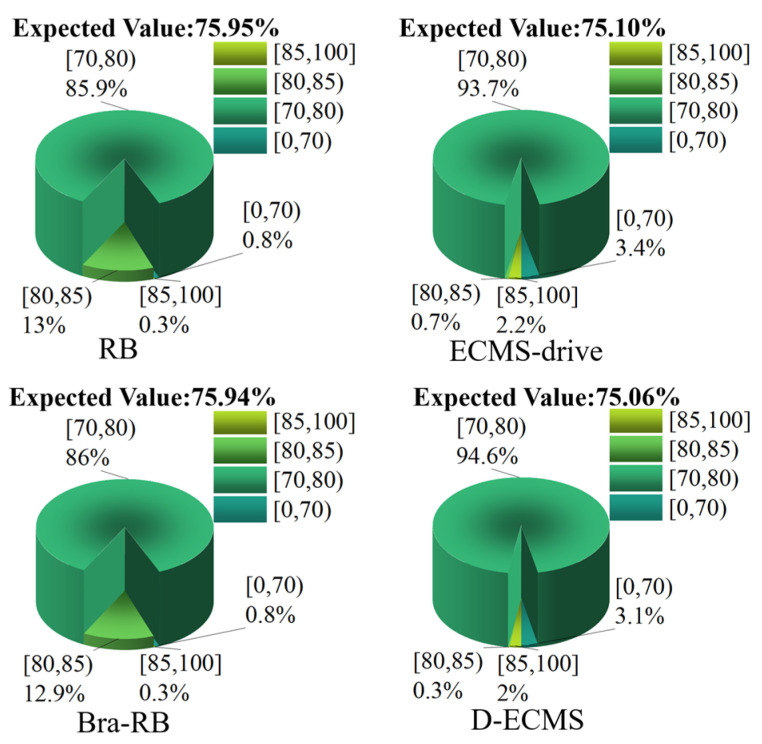
Efficiency distribution of front motor operating points in the driving process.

**Figure 12:**
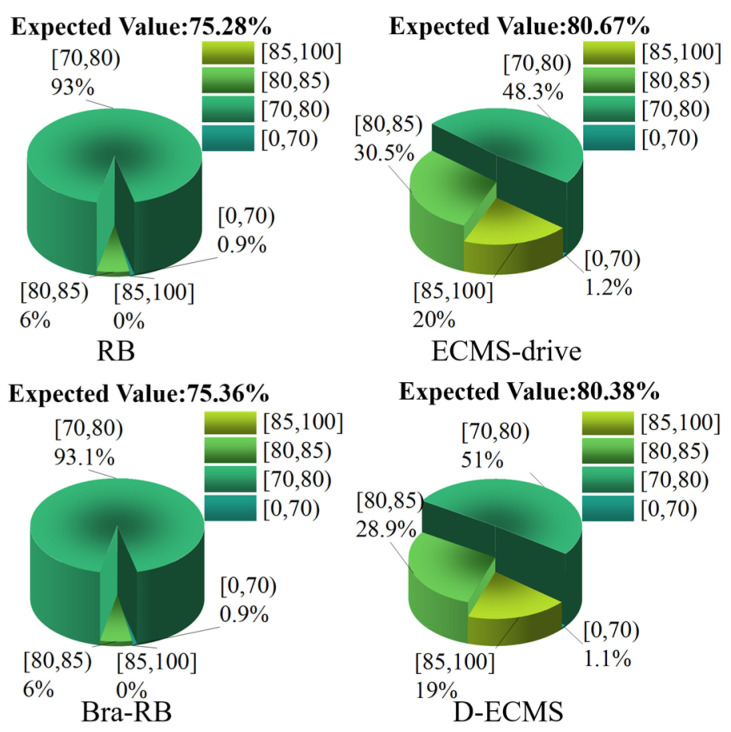
Efficiency distribution of rear motor operating points in the driving process.

**Figure 13:**
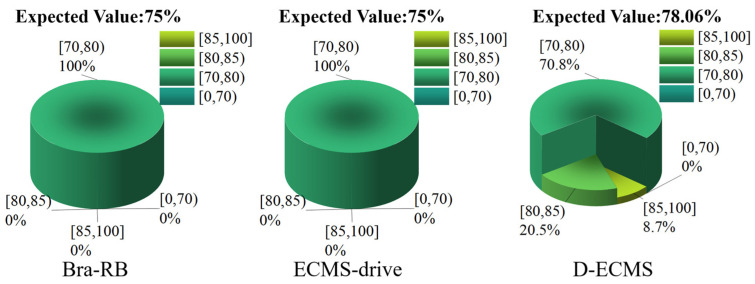
Efficiency distribution of front motor operating points in the braking process.

**Figure 14:**
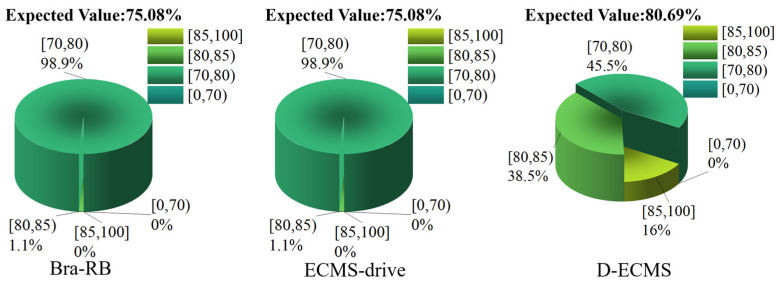
Efficiency distribution of rear motor operating points in the braking process.

In order to further analyze the influence of the control strategy on the efficient distribution of the front and rear motors’ operating points, this chapter divides the working efficiency of the front and rear motors into four different intervals, which are [85%,100%], [80%,85%), [70%,80%) and [0%,70%). Under different control strategies, the proportion of the operating points’ efficiency in the driving and braking process of the front and rear motors is explored. As shown in Figure 11, Figure 12, Figure 13 and Figure 14.

As shown in Figure 11, most of the operating points’ efficiency of the front motor under the four different controls are distributed in the interval [70%,80%), and the ratio of [80%,85%) in the efficiency interval based on RB and Bra-RB strategies is 13% and 12.9%, respectively. However, ECMS-drive and D-ECMS account for less in the efficiency interval [80%,85%), which are 0.7% and 0.3%, respectively. However, the ratio in efficiency interval [85%,100%] is 2.2% and 2%, which is significantly improved compared with the RB and Bra-RB strategy in the efficiency interval [85%,100%]. It is difficult for different control strategies to explore the advantages and disadvantages of the economy through the proportion of different efficiency intervals. Therefore, the mathematical expectation value of the operating points efficiency of the front motor under different strategies is analyzed. The expected work efficiency based on RB, Bra-RB, ECMS-drive and D-ECMS strategies are 75.95%, 75.94%, 75.10% and 75.06%. Under different control strategies, the mathematical expectation of the working efficiency of the front motor is basically similar, so the energy-saving potential of the front motor is basically the same.

Figure 12 shows the proportion of the operating points of the rear motor in the same efficiency interval under different control strategies. Both RB and Bra-RB strategies account for 6% [80%,85%). However, the strategies based on ECMS-drive and D-ECMS accounted for 30.5% and 28.9% in [80%,85%), respectively. The working efficiency of back-click based on the RB and the Bra-RB strategy is below 85%, and the proportion is 0% in [85%,100%]. In contrast, ECMS-drive and D-ECMS account for 20% and 19% [85%,100%], respectively, and the efficiency of the rear motor operating points is significantly improved. In the mathematical expectation analysis, the expected value based on RB, Bra-RB, ECMS-drive and D-ECMS strategies are 75.28%, 75.36%, 80.67% and 80.38%.

Through the analysis and comparison of Figure 11 and Figure 12**,** in the driving process, the energy output distribution of front and rear motors based on ECMS-drive and D-ECMS can effectively improve the work efficiency of motors, and the vehicle economy is significantly improved. In order to explore the distribution of the operating points of the front and rear motors in the braking process, Figure 13 and Figure 14 show the proportion of the operating braking points of the front and rear motors in different efficiency intervals under different control strategies.

The RB strategy adopts a braking energy recovery system, so the efficiency distribution of operating braking points is not explored. As shown in Figure 13, the front and rear motors adopt a fixed proportion of torque output based on Bra-RB and ECMS-drive in the braking process, and the braking operating points efficiency of the front motor is distributed in [70%,80%). Based on D-ECMS, the braking energy distribution of the front and rear motors is adopted, and the efficiency of the front motor operating point is 20.5% and 8.7% in [80%,85%) and [85%,100%], respectively. The efficiency of the operating points is significantly improved. In the mathematical expectation analysis, the expected working efficiency based on Bra-RB, ECMS-drive and D-ECMS strategies are 75%, 75% and 78.06%, and the motor working efficiency based on D-ECMS improves by 3.06% compared with the Bra-RB and the ECMS-driver.

According to Figure 14, the operating points of the rear motor based on Bra-RB and ECMS-drive are concentrated in the efficiency interval of [70%,80%), accounting for 98.9%, while only 1.1% of the operating points are distributed in the efficiency interval of [80%,85%) during the braking process. Under the D-ECMS, the working efficiency of the rear motor is greatly optimized, and the operating points account for 16% in the efficiency interval [85%,100%]. In addition, the proportion of efficiency interval [80%,85%) increased to 38.5%. In the mathematical expectation analysis, the expected working efficiency based on Bra-RB and ECMS-drive is 75.08%, while the expected working efficiency based on D-ECMS is 80.69%. The operating points efficiency of the rear motor is significantly improved.

Through the above analysis, under different driving conditions, there is a specific difference in the working efficiency due to the different working characteristics of the front and rear 4WD EV motors. Optimizing the efficiency of the front and rear motors’ operating points is essential to improving vehicle economy. The D-ECMS proposed in this paper realizes the energy distribution of the front and rear motors under the driving conditions and braking conditions. D-ECMS optimizes the working efficiency of the front and rear point motors and improves the economy and driving range of the 4WD EV.

## 5. Conclusions

Aiming at the cooperative energy output of multi-power components in 4WD EV under different driving states, a novel energy distribution strategy of D-ECMS is proposed in this paper to realize the energy distribution output of power components. The D-ECMS strategy is adopted to realize the cooperative energy output among the multiple power components of the vehicle, and improve the economy of the vehicle while ensuring the power performance. The operating points of the front and rear motors are optimized to give full play to the energy-saving potential of 4WD EV. In order to verify the effectiveness of this method, MATLAB/Simulink was used to complete the vehicle simulation. Compared with the RB strategy, the economy of the D-ECMS strategy is improved by 4.35%.

However, in the energy co-distribution of the motor before and after the vehicle, only the current working condition information is considered, and the future working condition information is not considered. In future scientific research, the knowledge of reinforcement learning will be studied and applied to obtain future information, improve the adaptability of control strategies and further improve the economy of vehicles.

## Figures and Tables

**Table 1:** Vehicle and dynamic components parameters in the 4DW PHEV.

Parameter	Unit	Value
Vehicle Mass	kg	1580
Vehicle Maximum velocity	km/h	170
Wheel rolling radius	m	0.35
Frontal area	m^2^	1.8
Front motor maximum power	kW	137
Front motor maximum torque	Nm	219
Rear motor maximum power	kW	87
Rear motor maximum torque	Nm	230
Battery capacity	kWh	47.5
Battery rated voltage	V	365

**Table 2:** Description of different control strategies.

Control Strategy	Description of Control Strategy
RB	Under the driving state, the front and rear motors adopt a fixed proportion of energy output, and the vehicle has no braking energy recovery system.
Bra-RB	Under the driving and braking state, the front and rear motors adopt a fixed a proportion of energy output.
ECMS-drive	Under the driving state, ECMS is used for driving energy distribution of the front and rear motors. Under the braking state, the front and rear motors adopt a fixed proportion of the braking energy recovery system.
D-ECMS	Under the driving and braking state, ECMS is used for driving energy distribution of the front and rear motors.

**Table 3:** Comparison of the simulation results and the data under different strategies.

Control Strategy	Terminal SOC	Economy (Relative to RB)
RB	0.4846	
Ber-RB	0.4928	1.69%
ECMS-drive	0.4992	3.01%
D-ECMS	0.5057	4.35%

## Data Availability

Not applicable.
